# Case report: Clinical analysis and literature review of five cases of metastatic solid pseudopapillary tumor of the pancreas

**DOI:** 10.3389/fonc.2024.1386987

**Published:** 2024-10-10

**Authors:** Run Hu, Renjie Gui, Xi Nie, Huaxin Duan

**Affiliations:** ^1^ Department of Oncology, Hunan Provincial People’s Hospital, The First Affiliated Hospital of Hunan Normal University, Hunan Normal University, Changsha, Hunan, China; ^2^ Key Laboratory of Study and Discovery of Small Targeted Molecules of Hunan Province, Hunan Normal University, Changsha, Hunan, China; ^3^ Hunan Provincial Clinical Medical Research Center for Hepatobiliary and Pancreatic Tumors, Changsha, Hunan, China; ^4^ Changsha City Comprehensive Diagnosis and Treatment Technology Innovation Center for Hepatobiliary Tumors, Changsha, Hunan, China

**Keywords:** solid pseudopapillary neoplasm of the pancreas, diagnosis, imaging, immunohistochemistry, treatment

## Abstract

**Background:**

Solid pseudopapillary neoplasm of the pancreas (SPN) is a rare and low-grade malignant tumor. It mainly occurs in women of reproductive age, accounting for approximately 1-3% of all pancreatic tumors. SPN has a low incidence rate and is difficult to diagnose before surgery. Some cases may show local infiltration, but distant metastasis rarely occurs. Currently, there is no standardized treatment protocol for SPN.

**Patient and methods:**

We have collected clinical data from 5 patients with solid pseudopapillary neoplasm (SPN) of the pancreas who presented with distant metastasis at our hospital. This study retrospectively analyzes their clinical manifestations, imaging characteristics, pathological findings, and treatment outcomes. The aim is to summarize the clinical features of SPN with distant metastasis, thereby improving the diagnosis, treatment, and prognosis prediction of this disease. This study also reviews relevant literature.

**Results:**

The median age of the 5 patients was 32 years old, with a male-to-female ratio of 1:4. All patients underwent enhanced CT scans and were diagnosed with SPN through biopsy or surgical pathology. All 5 patients had liver metastases, and one patient had clavicular lymph node metastasis. Another patient had both lung and clavicular lymph node metastases. Three patients underwent curative surgery, one patient received chemotherapy combined with targeted immunotherapy and subsequently underwent TACE(Transcatheter arterial chemoembolization) and HAIC (Hepatic artery infusion chemotherapy) treatments due to progression. One patient received internal radiation therapy but experienced multiple relapses and eventually died due to complications. The follow-up period ranged from 7 to 53 months, with 2 patients succumbing to the disease.

**Conclusion:**

As a low-grade tumor, SPN has a low rate of distant metastasis, typically occurring in only 5%-15% of cases. These metastases often lack characteristic clinical symptoms. Diagnosis can only be confirmed after exclusion of other lesions through imaging and pathological examination. The primary treatment for metastatic SPN is curative surgery, which can lead to a favorable prognosis.

## 1 Introduction

Solid pseudopapillary neoplasm of the pancreas (SPN) is a rare exocrine tumor, accounting for less than 3% of pancreatic tumors (1). SPN predominantly affects young females (2) and often presents with nonspecific clinical symptoms such as abdominal discomfort, bloating, abdominal pain, and abdominal mass. Some cases are incidentally detected during routine medical examinations. Laboratory tests usually show no significant abnormalities, and tumor markers are often negative. Due to the lack of specific clinical symptoms and normal laboratory findings, the preoperative diagnosis of SPN is challenging, leading to a high misdiagnosis rate. Therefore, imaging examinations, such as computed tomography (CT) and magnetic resonance imaging (MRI), are commonly used (3). According to the World Health Organization (WHO) classification criteria in 2010, the following features indicate malignancy in SPN: invasion of peripancreatic or deep tissues, vascular invasion, neural invasion, distant metastasis, and tumor recurrence (4). The definitive diagnosis and differentiation between benign and malignant SPN require pathological and immunohistochemical analysis.EUS-FNA which is currently common practice as preoperative diagnostic approach of SPN, increases the diagnostic performance with only rare side effects. EUS-FNA is useful in cases of diagnostic doubt and can differentiate SPN from other solid/cystic nodular lesion of the pancreas (5).

Therefore, it is of great importance to study the clinical and pathological features of SPN and their relationship with tumor malignancy, enabling preoperative assessment of tumor behavior for improved clinical management of SPN.

## 2 Study subjects and methods

### 2.1 Study subjects

This study includes 5 patients admitted to our hospital from September 2017 to March 2023, who were ultimately diagnosed with SPN with distant metastasis (to the liver, lungs, or lymph nodes). Among the patients, 4 were female and 1 was male, with ages of 47, 17, 32, 45, and 20 years, respectively. All patients were of Han ethnicity. One patient had previously undergone pseudocyst resection of the pancreas 2 years ago and had regular follow-up examinations. In January 2020, this patient was admitted with a diagnosis of “splenic vein thrombosis for 1 week” and received anticoagulant therapy. Another patient had a history of hepatitis B for over 20 years. None of the patients had a history of hypertension, diabetes, long-term smoking, or alcohol consumption. The patient’s clinical data can be found in **Table 1**.

**Table 1:** Patient clinical data.

number	sex	age	metastatic	pancreatic tumor size(cm)	clinical manifestation	Previous medical history	treatment	disease recurrence or progression	follow-up(month)
Case 1	Female	47	Liver	2.8*2.5*2.5	Bloating	Previous pseudocyst resection surgery of the pancreas	2023.03:GEMOX+Sulfatinib+Zimberelimab 2023.06.05:Transarterial chemoembolization (TACE) + hepatic artery infusion chemotherapy (HAIC)	2023.05.25: Tumor progression reassessment	7
Case 2	Female	17	Liver, lung,supraclavicular lymph nodes	11*10*6.5	Pancreatic mass detected on examination after trauma	nothing special	2020.03.23: Resection of pancreatic mass + right hemihepatectomy + resection of right lung nodule + resection of left clavicular lymph node 2020.06.09: Cryotherapy for tumor ablation of liver segment 8	2020.06.08: Detected intrahepatic recurrence	43
Case 3	Female	32	Liver	8*6.5*6	Liver mass detected during physical examination	nothing special	2023.02.21: Resection of massive tumor in the right liver + resection of tumor in the tail of the pancreas	No recurrence yet	7
Case 4	Male	45	Liver,supraclavicular lymph nodes	6*6*5	abdominal bloating and pain,jaundice	20-year history of hepatitis B	2017.10.11: Iodine-125 seed implantation in the head of the pancreas + resection and biopsy of liver nodules 2018.7-2020.12 8 sessions of TACE(Transarterial chemoembolization) 2019.11.08: Radiopharmaceutical particle implantation in the left supraclavicular lymph node	2018.05.30: Detected intrahepatic recurrence 2019.10.14: Detected recurrent metastasis to the left supraclavicular lymph node	40
Case 5	Female	20	Liver	12*10.5*8	Pancreatic and hepatic masses detected on examination after trauma	nothing special	2019.05.07: Right hemihepatectomy + resection of the body and tail of the pancreas	No recurrence yet	53
1 (6)	Female	49	Liver	6*5*4.8	abdominal discomfort	nothing special	a distal pancreatectomy with splenectomy an extended left hemihepatectomy A radiofrequency ablation	No recurrence yet	36
2 (7)	Female	71	Liver	5*3.8*3.5	pancreatic mass detected during physical examination	nothing special	distal pancreatectomy in 2013 Partial liver resection in 2017	Intrahepatic recurrence was detected 18 months after partial hepatectomy.	66
3 (8)	Female	19	Liver Splenic	6.7*6.2*3.7	acute abdominal pain vomiting	nothing special	a distal pancreatectomy, splenectomy, and partial hepatectomy adjuvant chemotherapy with FOLFIRINOX for 4 cycles (3 weeks interval). (FOLFIRINOX: Folinic acid, 5-FU, Irinotecan, Oxaliplatin)	recurrence of liver metastasis was noted 4 months after surgery	lost followed up
4 (9)	Male	39	Lymph Node	7.4*4.2*3.7	pancreatic mass detected during physical examination	nothing special	pylorus-preserving pancreaticoduodenectomy	No recurrence yet	14
5 (10)	Female	16	Liver	7.9*6.4*4.6	abdominal discomfort	nothing special	initial pylorus-preserving partial duodenopancreatectomy, right hemicolectomy, resection and allografting of the portal vein and secondary resection of 12 liver metastases	No recurrence yet	18
6 (11)	Female	61	Liver peritoneum Lung	9.9 × 8.6	epigastric pain	nothing special	pylorus-preserving pancreaticoduodenectomy	Nine years after the surgery	110

### 2.2 Study methods

A retrospective analysis of the patients’ clinical data was conducted, including age, gender, clinical manifestations, auxiliary examinations, diagnosis, treatment plans, and prognosis outcomes. The characteristics of metastatic SPN were summarized and discussed.

### 2.3 Case 1

**Figure 1:**
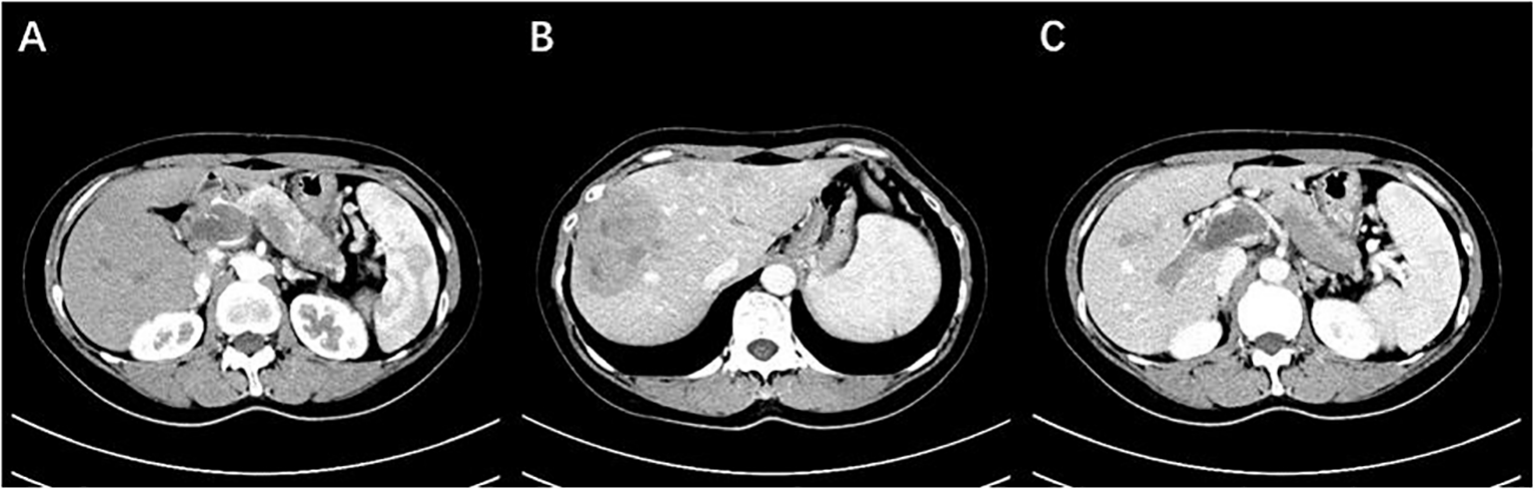
**(A)** The body and tail of the pancreas are enlarged, with indistinct borders, and show mild enhancement after contrast administration. **(B)** Multiple nodular lesions are observed within the liver, with uneven enhancement and low density. **(C)** Extensive filling defects with enhancement are observed in the portal vein, splenic vein, and superior mesenteric vein (Case 1).

A 47-year-old female patient presented in March 2023 with abdominal discomfort. She had a history of laparoscopic pseudocyst and jejunojejunostomy. Enhanced CT scan showed thickening of the body and tail of the pancreas, extensive thrombosis in the main portal vein and its branches, splenic vein, and superior mesenteric vein, as well as multiple liver metastases (**Figure 1**). Pancreatic and liver mass biopsies revealed solid pseudopapillary neoplasm of the pancreas with liver metastasis (**Table 2**). In March 2023, she started chemotherapy with gemcitabine and oxaliplatin for 6 cycles, along with immune checkpoint inhibitor therapy using Zimberelimab 300 mg once every 2 weeks and targeted therapy with Surufatinib 200mg daily. No severe adverse reactions were observed during treatment. In June 2023, reevaluation showed no reduction in the size of the pancreatic tumor, but progression of liver metastases. Subsequently, she underwent hepatic transarterial chemoembolization (TACE) with hepatic arterial infusion chemotherapy (HAIC) under local anesthesia. The medications used were gemcitabine 1.2g, oxaliplatin 100mg, cisplatin 40mg with iodized oil 20ml emulsion, leucovorin 300mg, and fluorouracil 2.5g. The arterial infusion chemotherapy lasted for 46 hours. The patient recovered well after the procedure and continued with oral Surufatinib targeted therapy. However, due to treatment ineffectiveness and tumor progression, she passed away in October 2023.

**Table 2:** Partial pathological and immunohistochemical results.

number	β-catenin	E-Cadherin	Vimentin	Syn	CD10	CgA	CD56	Ki67	CK(pan)	CK20	nerve/vessel invasion
1	(cytoplasm, nucleus+)	(-)	(+)	(+)	(weak+)	(-)	(+)	8%	(-)		uninfringed
2	(nucleus+)	(-)	(+)	(partial+)	(+)	(-)		5%	(+)	(-)	uninfringed
3	(diffuse+)	(-)	(diffuse+)	(diffuse+)	(diffuse+)	(-)		3%			infringement
4	(nucleus+)	(-)	(-)		(+)	(-)		30%	(+)	(-)	uninfringed
5	(+)	(-)		(+)		(-)	(+)	isolated positive	(+)	(-)	nerve invasion

Immunohistochemistry results are typically expressed as “+” for positive (i.e., the antigen is present) and "-" for negative (i.e., the antigen is absent or below the detection threshold).

### 2.4 Case 2

A 17-year-old female patient presented for the first time in December 2019 due to abdominal trauma. A comprehensive CT scan revealed a large mixed-density mass in front of the pancreas, indicating a tumor with associated bleeding. Multiple low-density lesions were also observed in the liver. Emergency exploratory laparotomy was performed under general anesthesia, including tumor resection in the retroperitoneum, partial reconstruction of the portal vein, liver mass biopsy, and adhesiolysis of the intestines. Postoperative pathology confirmed solid pseudopapillary neoplasm of the pancreas with liver metastasis. In March 2020, a PET-CT scan showed liver, right lung, and left clavicular lymph node metastases. Subsequently, the patient underwent single-port video-assisted thoracoscopic surgery for resection of the right middle lobe lung mass, open right hepatectomy with removal of the right lobe and tail, adhesiolysis of the intestines, and excision of the left clavicular lymph nodes under general anesthesia. Pathological examination confirmed metastasis of SPN. In June 2020, recurrent liver tumors were detected, and the patient underwent subsegmental liver ablation of the S8 metastatic lesion under local anesthesia. The patient has since been regularly followed up and no tumor recurrence has been observed. The follow-up duration is now 43 months.

### 2.5 Case 3

A 32-year-old female patient presented for the first time in February 2023 after a right liver mass was detected during a routine physical examination. There was no significant medical history. Laboratory tests showed no apparent abnormalities. Abdominal CT scan revealed a mass in the tail of the pancreas and multiple large cystic solid masses with hemorrhage in the liver. Subsequently, under general anesthesia, the patient underwent surgical resection of the large liver tumor, resection of the pancreatic tail tumor, splenectomy, cholecystectomy, bile duct reconstruction, exploration of the bile duct, and T-tube drainage. Postoperative pathology and immunohistochemistry confirmed the diagnosis of solid pseudopapillary neoplasm (SPN) with liver metastasis. The patient recovered well after the surgery and no tumor recurrence or metastasis has been observed during the 7-month follow-up period.

### 2.6 Case 4

A 45-year-old male patient initially sought medical attention in October 2017 due to abdominal distention and pain. He had a history of chronic hepatitis B for over 20 years. Laboratory results indicated elevated transaminases and bilirubin levels. A comprehensive abdominal CT scan revealed a mass in the tail of the pancreas and nodular lesions in the liver. Liver nodule resection biopsy confirmed the diagnosis of solid pseudopapillary neoplasm (SPN) with liver metastasis. Subsequently, the patient underwent pancreatic iodine-125 seed implantation. Seven months later, a follow-up examination showed tumor recurrence in the liver, leading to multiple transarterial chemoembolization (TACE) procedures. In October 2019, left clavicular lymph node metastasis was detected, and the patient underwent radioactive particle implantation in the left clavicular lymph nodes. During treatment, the patient experienced recurrent high fever, likely as a complication of TACE. The patient’s last visit was in December 2020, and telephone follow-up confirmed the patient’s death in February 2021. The total follow-up period was 40 months.

### 2.7 Case 5

**Figure 2:**
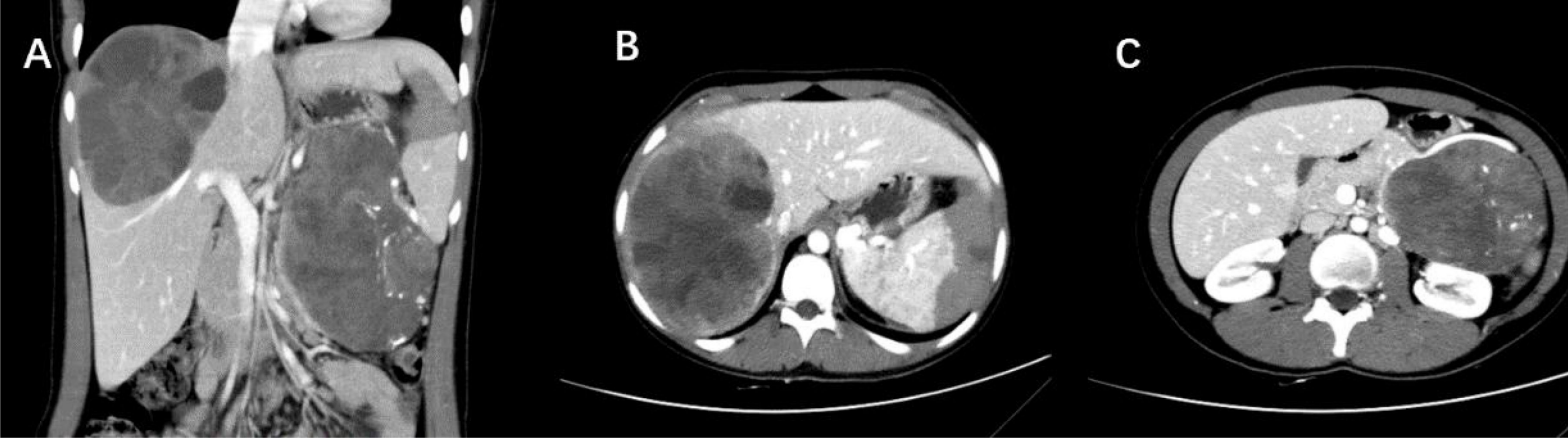
**(A)** CT scan shows a large, well-defined cystic-solid lesion in the tail of the pancreas and within the liver. **(B)** A massive oval-shaped lesion is observed in the right liver, with enhanced solid portions appearing as small patchy enhancements floating within the low-density cystic areas, demonstrating the “floating cloud sign.” **(C)** A cystic-solid mass is present in the tail of the pancreas, with intact capsule, and scattered calcifications seen within it (Case 5).

A 20-year-old female patient presented in April 2019 following trauma. A B-ultrasound examination revealed a mixed echogenic mass in the right lobe of the liver and the tail of the pancreas. Further evaluation with abdominal CT scan showed a large solid mass with calcification in the left retroperitoneum, causing compression and narrowing of the splenic artery, splenic vein, and left renal vein, as well as displacement of surrounding organs. Multiple lesions were also observed in the right liver, with compression and narrowing of the right hepatic vein (**Figure 2**). In May 2019, the patient underwent laparoscopic right hepatectomy, distal pancreatectomy, cholecystectomy, and splenectomy under general anesthesia. Postoperative pathology confirmed the diagnosis of solid pseudopapillary neoplasm (SPN) with liver metastasis. The patient reported feeling well after the surgery and has been regularly followed up. As of now, 53 months have passed since the surgery, and no tumor recurrence or metastasis has been observed.

## 3 Results

### 3.1 Clinical presentation

Among the 5 patients, 3 of them were asymptomatic and the pancreatic or liver masses were discovered during examinations. One patient complained of abdominal distension, and another patient reported upper abdominal pain with jaundice. All 5 patients exhibited varying degrees of tenderness in the upper abdomen during physical examination, with no rebound tenderness.

### 3.2 Laboratory tests

Blood routine, coagulation function, and amylase levels were all within normal ranges for the 5 patients. The results of some liver function tests and tumor markers are shown in **Table 3** below.

**Table 3:** Liver function and partial tumor marker results.

number	ALT(U/L)	AST(U/L)	TBIL(umol/L)	ALP(U/L)	CEA(ng/ml)	CA125(U/ml)	CA199(U/ml)
reference range	5-40	5-40	3.4-17.1	45-125	0-5	0-35	0-37
Case 1	26.8	30.8	34.9	64.6	2.58	8.30	18.41
Case 2	22.6	27.2	13.3	61	1.01	19.44	8.62
Case 3	17.9	30.6	14.19	41.6	0.59	13.8	11.94
Case 4	172.0	79.8	223.8	125	4.33	34.58	4.31
Case 5	25	21.2	5.9	122	1.56	21.57	30.25

### 3.3 Imaging examination results

All 5 patients underwent contrast-enhanced CT scans. In the cross-sectional images, the tumor appeared as cystic-solid in 4 cases and solid in 1 case. One patient exhibited calcification (**Figure 1**), and another patient showed signs of bleeding. Liver metastases were observed in all 5 patients, with 2 patients having lymph node metastasis in the supraclavicular region, 1 patient having lung metastasis, and 1 patient showing invasion of the portal venous system.

### 3.4 Pathological results

**Figure 3:**
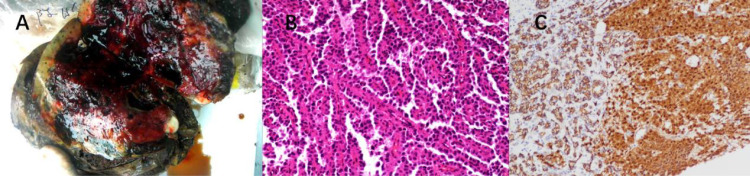
**(A)** Surgical specimen section of liver metastasis from SPN. **(B)** HE staining: Abundant solid nests of cells are visible, with rich small blood vessels. Tumor cells surround the small blood vessels, forming a so-called “pseudopapillary” arrangement. The nuclei of tumor cells show relatively uniform characteristics, and the cytoplasm exhibits acidophilia. **(C)** IHC: β-catenin staining shows nuclear/cytoplasmic positivity in tumor cells.

HE staining and immunohistochemical testing were performed on all 5 patients. Some immunohistochemical results are shown in **Table 2**. Some pathological results are shown in **Figure 3**. Based on the confirmed immunohistochemical results, all 5 patients were found to have liver metastasis. Among them, 1 patient had liver and supraclavicular lymph node metastasis, and another patient had liver, lung, and supraclavicular lymph node metastasis.

## 4 Discussion

Solid pseudopapillary neoplasm (SPN) is a rare type of pancreatic tumor, accounting for only 2% of exocrine tumors of the pancreas. SPN primarily occurs in female patients aged 20 to 40 years, with a distant metastasis rate of 1% to 5%. The most common sites of metastasis are the liver, peritoneum, and greater omentum, while reports of lung and lymph node metastasis are rare (12–14). In this study, a total of 5 patients with metastatic SPN were included, of which 4 were females.Of the relevant case reports we retrieved, 5 were female and 2 were male.The exact mechanism of SPN (oncogenesis) remains unclear. One theory suggests that SPN originates from pancreatic embryonic stem cells (15), while another viewpoint suggests its origin from the female reproductive system (16). Research has shown that more than 90% of SPN cases have mutations in the CTNNB1 gene (which encodes β-catenin). This mutation leads to the activation of the Wnt signaling pathway, preventing the degradation of β-catenin protein. Accumulated β-catenin can activate lymphoid enhancer binding factor (LEF) and T cell factor (TCF) in the nucleus, thereby upregulating the expression of various target genes. This ultimately promotes cell proliferation, differentiation, and epithelial-mesenchymal transition (17, 18). Furthermore, loss of E-cadherin expression on the cell membrane is also very common in SPN, and nuclear accumulation of β-catenin along with loss of E-cadherin membrane expression is often used to differentiate SPN from normal pancreas (19).

SPN lacks specific clinical symptoms, and most patients present with abdominal discomfort or a sense of bloating. A small portion of patients may experience nausea, vomiting, poor appetite, jaundice, and weight loss (12). Among the 5 patients included in this study, 3 patients were found to have pancreatic lesions during CT scans without any specific clinical manifestations. During the follow-up period of 2 patients who initially presented with abdominal bloating and pain, one female patient still experienced abdominal pain, suggesting a possible correlation with disease progression. The other male patient had recurring symptoms of abdominal pain and fever, which could be related to complications following TACE (Transarterial chemoembolization) treatment. In the literature we reviewed, the most common symptom is abdominal discomfort, with some also reporting vomiting (6–11).

Due to the lack of typical symptoms, accurate diagnosis and differentiation from other pancreatic diseases are crucial for SPN. The 5 patients included in this study showed no abnormalities in routine biochemical tests or tumor markers, thus diagnosis relied on imaging and pathology. SPN typically presents as oval or round cystic solid lesions within the pancreas or metastatic foci (20). The tumor is usually solitary, with a diameter typically exceeding 5cm. Based on the different proportions of cystic and solid components, it can be classified into predominantly cystic, predominantly solid, or equal cystic and solid types, with the latter being more common (21). In cases where the solid component is predominant, SPN appears as nodular or mass-like low-density shadows on plain scans, and shows progressive enhancement on contrast-enhanced scans. However, the degree of enhancement is always lower than that of normal pancreatic parenchyma, which is a typical characteristic of SPN. In predominantly cystic or equally cystic and solid SPN, the solid part shows patchy enhancement or floats within the low-density cystic part, which can be referred to as the “floating cloud sign” (22). Another imaging characteristic of SPN is the presence of a capsule. The outer wall of the capsule is usually smooth, enhanced after contrast administration, and has a clear demarcation from the pancreas and surrounding tissues. The integrity of the capsule can serve as an important imaging basis for evaluating the malignancy of the tumor (23). Additionally, calcification can occur in some cases, mainly located at the margin of the tumor, presenting as punctate, patchy, or arc-shaped calcifications on imaging. This is also an important sign for diagnosing SPN (24). MRI, due to its higher soft tissue resolution and multi-planar, multi-parameter imaging capabilities, has advantages in displaying hemorrhage, capsule, and internal cystic-solid changes (25).

Pathology remains the gold standard for diagnosing SPN. Under the microscope, a mixture of solid and pseudopapillary structures can be observed along with areas of hemorrhage and pseudocystic changes. The solid nests of cells are abundant, surrounded by rich small blood vessels. Cells further from the blood vessels have low adhesion and tend to detach, while cells closer to the blood vessels form pseudopapillary structures around the fibrovascular axis. The fibrovascular axis may exhibit hyaline or mucinous degeneration. There may be cytoplasmic vacuoles, but no true glandular lumens. The cells are relatively uniform, well-differentiated, and have moderately acidophilic cytoplasm. The nuclei often show nuclear grooves or twists, with evenly distributed chromatin and inconspicuous nucleoli. Occasionally, bizarre nuclei may be seen, but mitotic figures are rare (26). Typical tumor cells may contain small transparent droplets that stain positive for periodic acid-Schiff (PAS) after digestion. In some cases, cholesterol crystals can be seen, surrounded by foreign-body giant cells and foam cells, which can undergo calcification or even ossification. The tumor usually has clear borders, but focal invasion of surrounding pancreatic tissue and rare vascular and neural invasion can occur. SPN exhibits a diverse immunophenotype, with the most prominent immunohistochemical feature being the simultaneous expression of β-catenin in the cytoplasm and nucleus (27). E-cadherin is typically absent in membrane expression, possibly related to β-catenin, which serves as another important diagnostic clue (28). Other positive markers include α1-antitrypsin (AAT), Cyclin D1, Vimentin, CD10, CD99 (dot-like positivity), CD56, and Claudin-7, among others. Additionally, Syn and NSE may show focal positivity, while CgA is generally negative (29). Negative immunohistochemical markers include EMA, CEA, AFP, CA19-9, and others.

Overall, SPN has a favorable prognosis, with over 95% of patients achieving cure after surgery and a high quality of survival, with disease-free survival exceeding 10 years (30–32). As a low-grade malignancy, SPN rarely metastasizes. Studies have shown that tumor diameter >8 cm, vascular invasion, and Ki67 >1% are high-risk factors for recurrence and metastasis of SPN (13, 14). Surgical resection is the preferred treatment for SPN, and the majority of patients experience good outcomes after surgery. Even for patients with synchronous or metachronous distant metastasis or recurrence, surgery can still achieve favorable treatment effects (33–36). In this study, 3 patients underwent radical surgery for the primary and metastatic lesions. In the follow-up period, 2 patients remained disease-free, while 1 patient experienced liver tumor recurrence 2 months after surgery but remained disease-free after ablation. Another male patient underwent pancreatic head radiotherapy and surgical resection of liver metastases. During the follow-up, liver tumor recurrence and lymph node metastasis were detected, and lymph node radioembolization was performed. However, the patient repeatedly experienced high fever, indicating liver abscess caused by postoperative infection. The patient died 2 months after the last TACE procedure. There is no standard systemic treatment for unresectable, recurrent, or metastatic SPN. Individual clinical cases have reported the use of different chemotherapy regimens, such as gemcitabine, fluorouracil, cisplatin, paclitaxel, etc. (37–39). In addition, targeted therapies with multi-target receptor tyrosine kinase inhibitors like sunitinib, mTOR inhibitors like everolimus, and COX-2 selective inhibitors like celecoxib have been reported (40–42). Endocrine therapy with tamoxifen and other treatments can be attempted for ER/PR-positive patients (43). Radiation therapy has also been used in some cases of SPN (44–46), but its effectiveness still requires further validation. In this study, one female patient received chemotherapy with the GEMOX regimen combined with sunitinib targeted therapy and immunotherapy with cetuximab. After 6 cycles of treatment, tumor progression was detected. Subsequently, TACE+HAIC treatment was performed due to liver tumor rupture. However, the patient eventually died due to ineffective targeted therapy.

## 5 Conclusion

In summary, SPN is a low-grade tumor, and surgical treatment is the first choice. Even in cases of recurrence or metastasis, favorable outcomes can still be achieved after surgical resection. Additionally, adjuvant chemotherapy, regional chemotherapy infusion, local radiation therapy, targeted therapy, and endocrine therapy can also benefit patients to some extent.

## Data Availability

The datasets presented in this study can be found in online repositories. The names of the repository/repositories and accession number(s) can be found below: https://www.ncbi.nlm.nih.gov/, 1.
